# *Drosophila *type II neuroblast lineages keep Prospero levels low to generate large clones that contribute to the adult brain central complex

**DOI:** 10.1186/1749-8104-5-26

**Published:** 2010-10-01

**Authors:** Omer Ali Bayraktar, Jason Q Boone, Michael L Drummond, Chris Q Doe

**Affiliations:** 1 Institute of Molecular Biology, Howard Hughes Medical Institute, University of Oregon, Eugene, Oregon 97403, USA; 2 Institute of Neuroscience, Howard Hughes Medical Institute, University of Oregon, Eugene, Oregon 97403, USA

## Abstract

Tissue homeostasis depends on the ability of stem cells to properly regulate self-renewal versus differentiation. *Drosophila *neural stem cells (neuroblasts) are a model system to study self-renewal and differentiation. Recent work has identified two types of larval neuroblasts that have different self-renewal/differentiation properties. Type I neuroblasts bud off a series of small basal daughter cells (ganglion mother cells) that each generate two neurons. Type II neuroblasts bud off small basal daughter cells called intermediate progenitors (INPs), with each INP generating 6 to 12 neurons. Type I neuroblasts and INPs have nuclear Asense and cytoplasmic Prospero, whereas type II neuroblasts lack both these transcription factors. Here we test whether Prospero distinguishes type I/II neuroblast identity or proliferation profile, using several newly characterized Gal4 lines. We misexpress *prospero *using the 19H09-Gal4 line (expressed in type II neuroblasts but no adjacent type I neuroblasts) or 9D11-Gal4 line (expressed in INPs but not type II neuroblasts). We find that differential *prospero *expression does not distinguish type I and type II neuroblast identities, but Prospero regulates proliferation in both type I and type II neuroblast lineages. In addition, we use 9D11 lineage tracing to show that type II lineages generate both small-field and large-field neurons within the adult central complex, a brain region required for locomotion, flight, and visual pattern memory.

## Introduction

*Drosophila *neural progenitors, called neuroblasts (NBs), are an excellent model system to study progenitor self-renewal and differentiation mechanisms [1]. NBs divide asymmetrically to generate a larger self-renewing NB and a smaller differentiating progeny. Genetic analyses have identified proteins partitioned into the NB that promote self-renewal and proteins partitioned into the smaller progeny that promote differentiation [2].

Recent work has shown that there are two types of NBs in the *Drosophila *larval brain: type I and type II [3-5]. There are approximately 90 type I NBs per brain lobe that have nuclear Deadpan (Dpn), nuclear Asense (Ase), and cytoplasmic Prospero transcription factors. They divide asymmetrically to bud off small ganglion mother cells (GMCs) that undergo a terminal symmetric division to produce two neurons [1]. Type I NBs express all known apical/basal polarity markers. Apical markers are segregated into the NB, where they can promote aspects of NB identity [6]; basal markers such as Miranda, Prospero, Brain tumor (Brat), and Numb are segregated into the GMC, where they promote neuronal differentiation [7-11]. Axons formed by the neuronal progeny of central brain type I lineages fasciculate with each other and generally project within a single stereotyped tract to their targets [12]; this is different from type I NB lineages in the ventral nerve cord, which exhibit axon branching [13].

**Figure 1:**
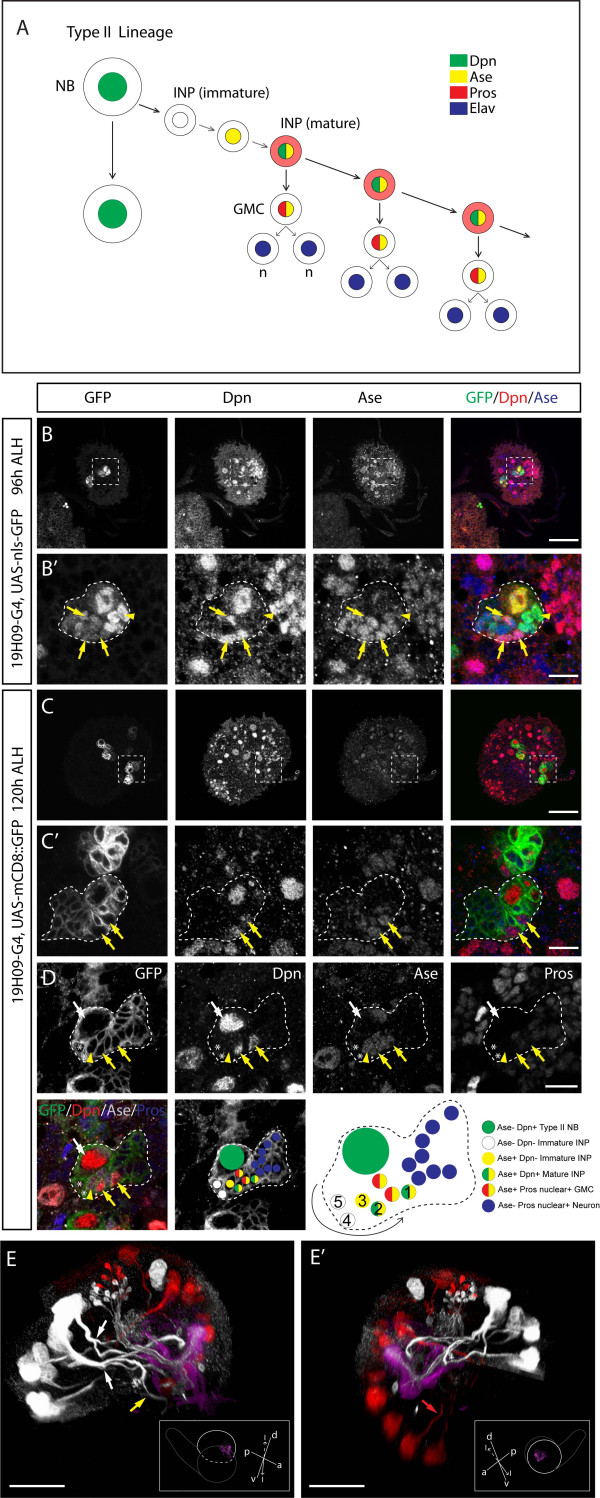
**19H09-Gal4 labels a subset of type II neuroblasts and their progeny**. **(A) **Type II NB lineage summary, modified from [4]. **(B-D) **Confocal images of third instar larval brains expressing nls::GFP (B) or mCD8::GFP (C) under 19H09-Gal4 stained for indicated markers (white box). Low magnification images of single brain lobes are presented in (B,C) and high magnification images of boxed areas are presented in (B',C'), respectively. (D) A high magnification image of a type II NB and associated progeny from a different brain. The white dotted outlines represent the green fluorescent protein (GFP)-labeled areas. Type II NB are large Dpn+ Ase- cells (white arrows). Ase- Dpn- immature INPs next to type II NBs are indicated with asterisks, Ase+ Dpn- immature INPs close to type II NBs are indicated with arrowheads and mature INPs are indicated with yellow arrows. The type II lineage shown in (D) is diagrammed below indicating the different kinds of cells in the lineage. The birth order of the cells was inferred from their relative position to the parental NB: newly born cells (5) are in direct contact with the type II NB while earlier-born cells (1) and their progeny are displaced and found further away. **(E,E') **Three-dimensional reconstruction of medial (E) and lateral (E') views of a 120 h ALH (after larval hatching) brain lobe expressing mCD8::GFP under control of 19H09-Gal4. Type II lineages and their axonal projections are in white, the mushroom body, visualized by FasII, in magenta, and type I lineages and their projections in red. Additionally, a subset of neurons that project to the mushroom body are visualized by the driver. The optic lobes have been removed and the brain cropped for a clearer view. Brains (gray outline) are in the orientations shown in the insets, with imaged lobes indicated with a white dashed line and their mushroom bodies shown. The rest of the brain apart from the imaged lobes is indicated in white outline. Split axon tracts of type II lineages are indicated with white arrows, the yellow arrow points at a commissural projection from a type II lineage, and the red arrow points at a type I projection. Orientation: d, dorsal; v, ventral; p, posterior; l, lateral; m, medial. Scale bars: (B,C) 50 μM; (B',C',D) 10 μM; (E,E') 40 μM.

There are only eight type II NBs per brain lobe, and they can be identified as large Dpn+ cells that are Ase- Prospero- (unlike type I NBs). Type II NBs express all known apical/basal polarity markers except for Prospero, and they bud off small progeny that lack Prospero protein. These type II NB progeny have been called transit amplifying GMCs [4], intermediate progenitors [3], or secondary NBs [5]. Here we will use the term intermediate neural progenitors (INPs) because it accurately reflects the position of these cells within the lineage (intermediate between NB and GMC) and the proliferation ability (intermediate between NB and GMC), and it is less likely to be confused with either NB or GMC cell types. Each INP divides between four and eight times to generate equal-sized siblings: another INP and a GMC that produces a pair of neurons (Figure 1A). Due to the extended proliferation of the INPs, each type II NB contributes a far larger population of neurons to the adult fly brain compared to a type I NB [3-5].

Recently, type II lineages have been shown to be susceptible to tumor formation: loss of the translational repressor Brat or the Notch repressor Numb or the transcription factor Earmuff from the whole brain results in tumor formation only within type II lineages [5,9,14]. Tumor formation is due to INPs reverting back to a type II NB-like identity; interestingly, the tumor phenotype can be suppressed by ectopic Prospero [5,9,14]. This raises the possibility that Prospero overexpression suppresses *brat *or *numb *tumors by transforming type II NBs to a type I NB identity. Consistent with this model, only type I NBs contain detectable levels of Prospero protein - type II NBs lack Prospero protein [3-5]. Alternatively, Prospero could inhibit proliferation in type II NBs without altering their cell fate. Consistent with this model, loss of *prospero *from embryonic or larval type I NB lineages leads to failure to repress cell cycle genes [15,16] and 'tumor' formation [5,9,11,14]. Similarly, the Prox1 vertebrate ortholog is expressed in newly differentiating neurons [17], inhibits neural progenitor proliferation [18], and is a candidate tumor suppressor gene [19-21].

Here we characterize two Gal4 lines that allow us to manipulate *prospero *expression within type II NBs and their INP progeny. We use these lines to test whether Prospero controls the difference between type I and type II NB identity, or whether it acts to limit progenitor proliferation without affecting NB identity. In addition, we use these lines to perform heritable lineage tracing to determine, for the first time, the adult brain neurons generated by the type II NB lineages.

## Results

### Identification of 19H09, a Gal4 line expressed in type II neuroblasts and INPs

To identify Gal4 lines that would allow us to manipulate Prospero expression in type II lineages and INPs, we screened Gal4 lines available from public stock centers and enhancer-Gal4 lines targeted to the attP third chromosomal location (Manning *et al*., unpublished results) [22]. Here we describe the 19H09-Gal4 line, which is expressed in five to seven out of the eight type II NBs and their INP progeny. The line is also expressed in a few type I NBs (which are ventral and thus easy to exclude from our analyses) and some post-mitotic neurons that project to the mushroom body (Figure 1E,E'; Additional file 1).

We analyzed 19H09 expression at 24, 48, 72, 96 and 120 h after larval hatching (ALH) by driving expression of nuclear green fluorescent protein (GFP). We observed no brain expression from 24 to 72 h ALH (data not shown). At 96 h and 120 h ALH we observed expression in five type II NBs and numerous adjacent small cells (Figure 1B; Table 1). We identify the NBs as type II based on their lack of Ase and ability to generate small Ase+ Dpn+ progeny [3-5]. As expected, the 19H09-labeled type II progeny include the newly born 'immature' INPs that have not yet upregulated Dpn [5] (Figure 1D, asterisks). However, some of these immature Dpn- INPs were Ase+, showing that Ase is upregulated prior to Dpn during INP maturation (Figure 1B',D). More distant from the parental type II NB were the mature Dpn+ Ase+ INPs and the Prospero+ GMCs derived from each INP (Figure 1D). Thus, analysis of 19H09 expression confirms that type II NBs generate INPs, and shows for the first time that INPs mature by upregulating Ase followed by Dpn, prior to dividing to produce GMCs.

**Table 1:** Analysis of 19H09 expression in wild-type and *prospero *misexpression brains

Genotype and stage	**Type II NB**^**a**^	**GFP+ type II NB**^**b**^	**GFP+ type I NB**^**c**^	**GFP+ INP**^**d**^	**GFP+ type II progeny**^**e**^	**Sample size**^**f**^
19H09-G4, UAS-nls-GFP @ 25°C						
96 h ALH	8.0	4.6 ± 0.5	1.9 ± 1.3	18.9 ± 5.9	58.9 ± 17.4	7
120 h ALH	8.0	5.4 ± 0.5	6.6 ± 1.9	62.9 ± 6.4	138.1 ± 14.1	8
19H09-G4, UAS-mCD8::GFP @ 25°C						
96 h ALH	8.0	5.5 ± 0.7	1.9 ± 1.4	31.7 ± 5.9	84 ± 16.3	10
120 h ALH^g^	8.0	6.5 ± 0.8	8.3 ± 1.5	81.9 ± 9.0	232.5 ± 14.2	8
19H09-G4, UAS-mCD8::GFP @ 30°C						
120 h ALH^g^	8.0	5.8 ± 0.4	7.0 ± 1.8	75.5 ±3.7	242.7 ± 34.5	6
19H09-G4, UAS-mCD8::GFP, UAS-pros @ 30°C						
120 h ALH	7.3 ± 0.5	4.6 ± 0.7	7.3 ± 1.5	9.8 ± 2.6	108.8 ± 13.5	9

^a^Large Dpn+ Ase-. ^b^Large GFP+ Dpn+ Ase-. ^c^Large GFP+ Dpn+ Ase+. ^d^Small GFP+ Dpn+. ^e^Small GFP+ cells in type II lineages. ^f^Brain lobes. ^g^At 120 h ALH, 19H09-driven mCD8::GFP weakly labels one or two extra type II lineages.

As further confirmation that 19H09 drives expression in type II NBs and their progeny, we drove expression of a membrane-tethered GFP to trace axon projections (Figure 1C-E). We observe immature and mature INPs adjacent to the type II NBs (Figure 1C) as well as the projections of the earlier-born neurons in the lineages (Figure 1E,E'; Additional file 1). We observed that some of these secondary axon tracts were split and targeted towards different parts of the brain (Figure 1E, white arrows) unlike type I axon projections in the central brain, which generally extended along a single tract (Figure 1E', red arrow) [12]. We also observed commissural projections from type II lineages (Figure 1E, yellow arrow). Since 19H09 is not expressed before 72 h ALH, only a subset of secondary axon projections of type II lineages were labeled. Our observations confirm and extend the findings from clonal analysis of type II lineages [23]. We conclude that 19H09 can be used to drive gene expression in type II NBs and their INP, GMC, and neuronal progeny beginning at late larval stages.

### Identification of 9D11, a Gal4 line expressed in INPs and their progeny

**Figure 2:**
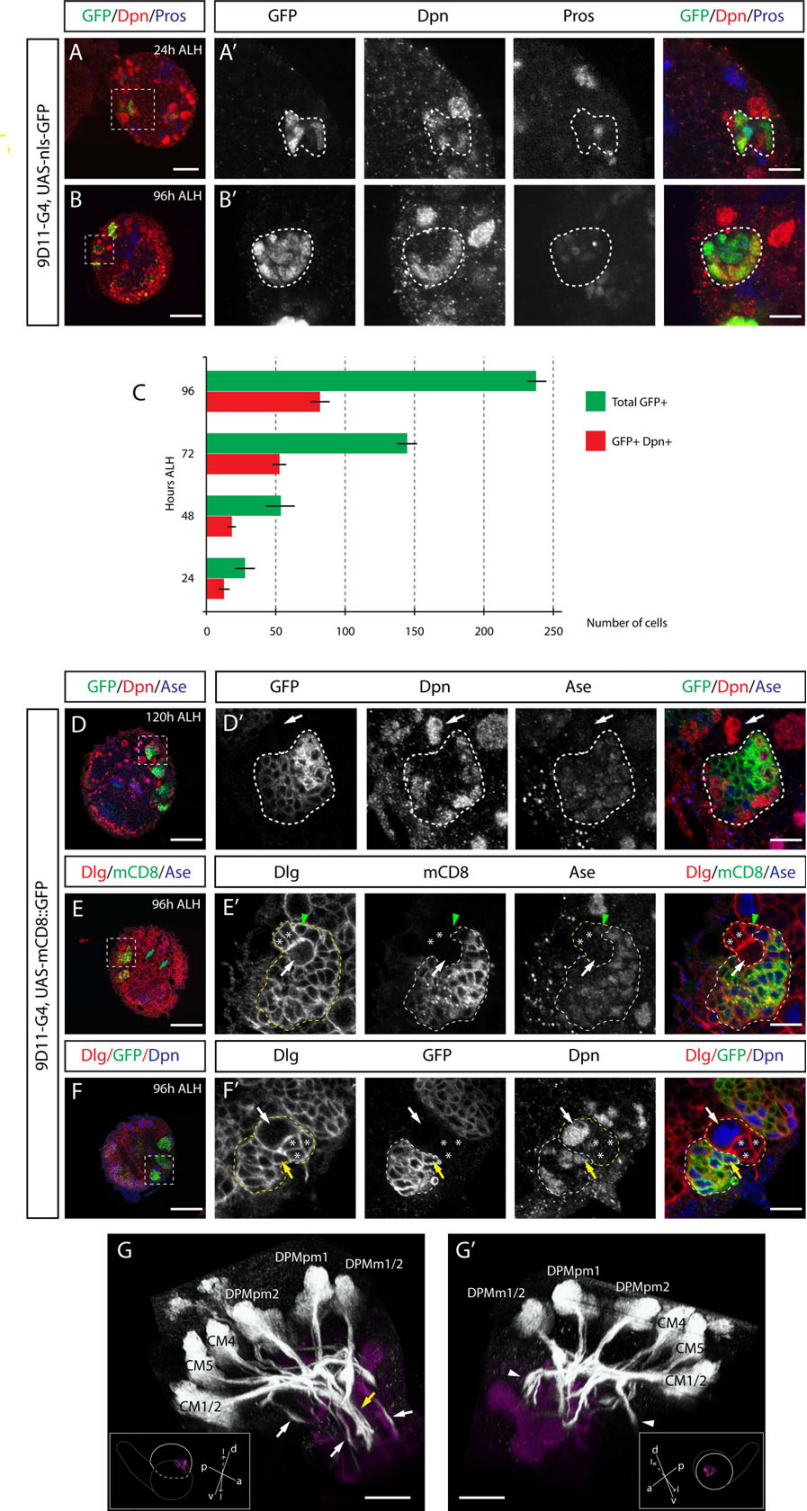
**9D11 specifically labels INPs and their progeny within the central brain**. **(A,B,D-F) **Confocal images of first (A) and third (B,D,E,F) instar larval brains expressing nls-GFP (A,B) or mCD8::GFP (D-F) under 9D11-Gal4 stained for indicated markers (white boxes). Low magnification images of single brain lobes are presented in (A-F), and high magnification images of boxed areas are presented in (A'-F'), respectively. White outlines represent the GFP-labeled areas; yellow outlines represent NB lineages visualized by Discs-large (Dlg) staining. Type II NBs are indicated with white arrows; Ase- Dpn- immature INPs next to type II NBs are indicated with asterisks; Ase+ Dpn- immature INPs close to type II NBs are indicated with arrowheads. Mature INPs are small Dpn+ cells in white outlined areas or indicated with yellow arrows. (E) Green arrows point at the two lateral type II NBs. **(C) **Histogram showing increasing number of INPs and total cells labeled by nls-GFP driven by 9D11-gal4 between 24 and 96 h ALH. Error bars indicate standard deviation. **(G,G') **Three-dimensional reconstruction of medial (G) and lateral (G') views of a 120 h ALH brain lobe expressing mCD8::GFP under control of 9D11-Gal4. Type II lineages and their axonal projections are shown in white, and the mushroom body, visualized by FasII, is shown in magenta. The optic lobe is removed and the brain cropped for a clearer view. See text for lineage labels. Brains (gray outline) are in the orientations shown in the insets, with imaged lobes indicated with a white dashed line and their mushroom bodies shown, same as in Fig. 1. White arrows point at commissural projections and arrowheads point at descending ipsilateral projections from type II lineages. The yellow arrow points at the dorsoposterior commissure. Type II lineages were labeled according to [12]. Orientation: d, dorsal; v, ventral; p, posterior; l, lateral; m, medial. Scale bars: (A) 20 μM; (B-F) 50 μM; (A'-F') 10 μM; (G,G') 40 μM. CM, centromedial lineages. DPMm, medial dorsoposterior lineages, medial subgroup. DPMpm, medial dorsoposterior lineages, posteromedial subgroup.

The 9D11-Gal4 line was generated by fusing *cis*-regulatory DNA from the *earmuff *gene to Gal4 [22]; it shows expression in the dorsomedial and centromedial larval brain region with axon projections similar to those shown for type II NB progeny (compare Image 3 in [22] with Image 2 in [4] and Images 2 to 7 in [23]). We found that 9D11-Gal4 is specifically expressed in an increasing number of INPs from 24 h to 96 h ALH (Figure 2A-C; Table 2) but not in the type II NB (Figure 2D'-F', white arrows). In the type II lineages, mature INPs (Figure 2F', yellow arrow) but not the Ase- and Ase+ immature INPs (Figure 2E',F', asterisks and arrowhead, respectively) were labeled, showing that 9D11 expression correlates with INP maturation. The absence of 9D11 expression from immature INPs is consistent with previous observations [14]. No other cells in the central brain expressed 9D11, but expression was found in the optic lobe (data not shown). We confirmed that 9D11 is expressed in INPs by crossing it to UAS-mCD8::GFP and observing axon projections that match the previously identified axon projection pattern of type II progeny (Figure 2G,G'; Additional file 2) [4,23]. 9D11 is strongly expressed in INPs within the six medial type II lineages (Figure 2G,G'); these are likely to be the DM1-DM6 NBs) [12,23]. 9D11 is also expressed weakly in the remaining two lateral lineages beginning at 96 h ALH (Figure 2E,G,G').

**Table 2:** Analysis of 9D11 expression in wild-type and *prospero *misexpression brains

Genotype and stage	**GFP+ INP**^**a**^	**GFP+ type II progeny**^**b**^	**Sample size**^**c**^
9D11-G4, UAS-nls-GFP @ 25°C			
24 h ALH	12.4 ± 3.5	27 ± 5.8	8
48 h ALH	18.1 ± 2.4	52.6 ± 9.7	8
72 h ALH	51.7 ± 4.4	142.2 ± 6.6	13
96 h ALH	80.1 ± 6.2	232.7 ± 6.2	10
9D11-G4, UAS-mCD8::GFP @ 25°C			
24 h ALH	11.8 ± 5.8	25.6 ± 5.8	5
48 h ALH	16.11 ± 5.5	53.44 ± 18.3	8
72 h ALH	52.1 ± 10.3	527.5 ± 24.4	9
96 h ALH	83.5 ± 2.5	548.9 ± 14.9	7
9D11-G4, UAS-mCD8::GFP @ 30°C			
96 h ALH	86.6 ± 6.3	619.3 ± 20.1	7
120 h ALH	710.8 ± 14.1	97.6 ± 2.3	5
9D11-G4, UAS-mCD8::GFP, UAS-Pros @ 30°C			
96 h ALH	14.2 ± 2.8	197.6 ± 20.6	10
120 h ALH	18.6 ± 2.8	214.8 ± 18.3	8

^a^Small GFP+ Dpn+. ^b^Small GFP+. ^c^Brain lobes.

As in the case with 19H09, the type II axon projections labeled with 9D11 were split into several branches and targeted towards different parts of the brain (Figure 2G,G'; Additional file 2). These projections included commissural (Figure 2G, arrows) and descending ipsilateral (Figure 2G', arrowheads) bundles; the former were observed from all six medial type II lineages. Type II axonal fibers entered the larval commissure at different sites but a significant portion of labeled projections were targeted to the dorsoposterior commissure (DPC; Figure 2G, yellow arrow), which is a part of the larval precursor to the central complex of the pupal brain [12]. Upon labeling with 9D11 it was difficult to trace trajectories due to dense staining, yet we were still able to individually identify 9D11+ type II lineages by the positional information of cell body clusters (that is, stereotypical anterior-to-posterior arrangement of the medial lineages) and by matching the visible projections to previous data (Figure 2G,G', lineages labeled) [12,23]. We conclude that the medial type II lineages make complex secondary axon projections and project a subset of their axons to the interhemispheric commissure.

### Prospero misexpression suppresses proliferation in type II NBs but does not induce type I NB identity

**Figure 3:**
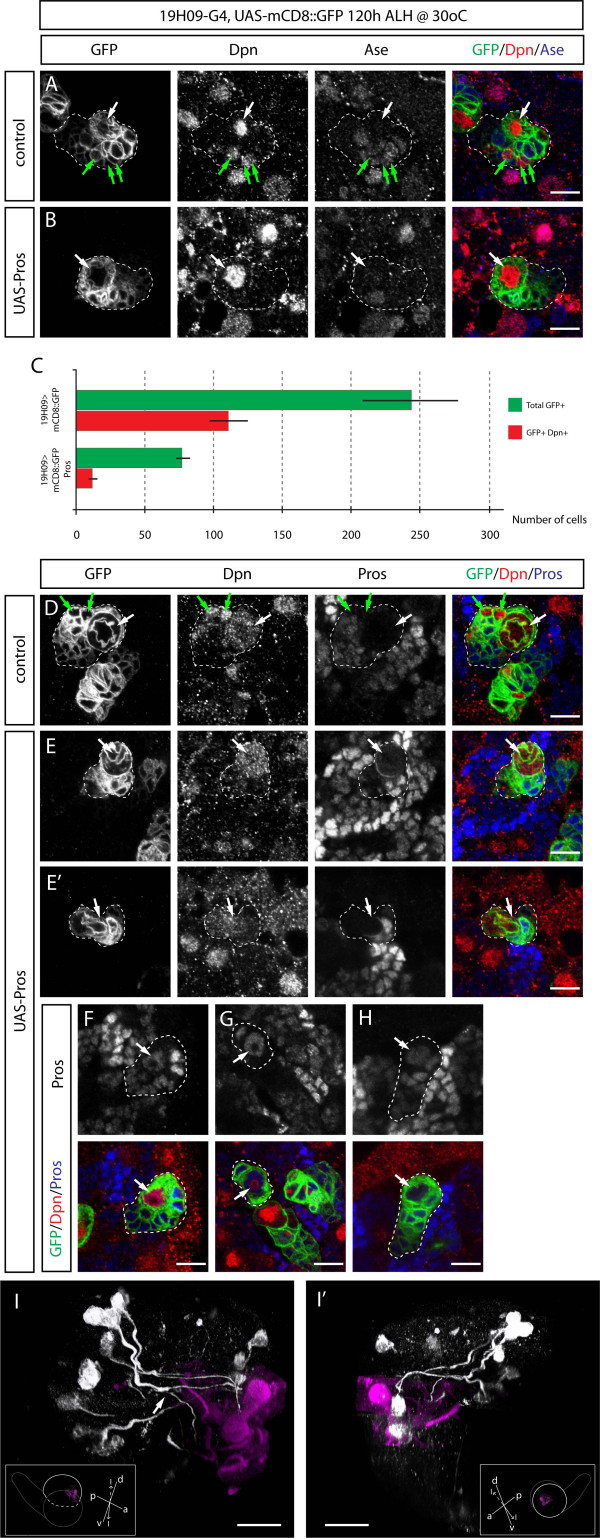
**Prospero misexpression in type II NBs reduces lineage size but does not induce type I NB identity**. **(A,B,D-H) **High magnification confocal images of type II NBs and associated progeny from third instar larval brains expressing mCD8::GFP (A,D) or mCD8::GFP and Pros (B,E-H) under control of 19H09-Gal4. White outlines represent the GFP labeled areas. Type II NBs are indicated with white arrows and mature INPs are indicated with green arrows. **(C) **Histogram showing number of INPs and total cells labeled by mCD8::GFP driven by 19H09-gal4 in control and *pros *overexpression brains at 120 h ALH. Error bars indicate standard deviation. **(I,I') **Three-dimensional reconstruction of medial (I) and lateral (I') views of a 120 h ALH brain lobe expressing mCD8::GFP and Pros under control of 19H09-Gal4. Type II lineages and their axonal projections are showm in white, and the mushroom body, visualized by FasII, is shown in magenta. The optic lobe is removed and the brain cropped for a clearer view. Brains are in the orientations shown in the insets, with imaged lobes indicated with a white dashed line and their mushroom bodies shown. The white arrow points at a split axon tract from a type II lineage. Orientation: d, dorsal; v, ventral; p, posterior; l, lateral; m, medial. Scale bars: (A-E) 10 μM; (F,F') 40 μM.

After characterizing the type II and INP Gal4 lines 19H09 and 9D11, we next used these lines to test whether misexpression of Prospero could induce a type II to type I NB transformation. Type I NBs contain cytoplasmic Prospero at interphase, form Prospero basal cortical crescents during mitosis, and generate only nuclear Prospero+ GMCs that undergo a terminal cell division. In contrast, type II NBs lack detectable Prospero protein at all stages of the cell cycle and generate nuclear Prospero- INPs that can divide multiple times [3-5]. We used the 19H09-Gal4 line to drive low levels of Prospero in type II NBs, and observed cytoplasmic Prospero at interphase and basal cortical Prospero at mitosis (100%, n = 11 mitotic NBs; Figure 3), similar to type I NBs [3-5]. However, the NBs remained Ase- and generated bifurcating axon projections characteristic of type II NBs in the central brain (Figure 3B,I; Additional files 3 and 4). Expression of higher levels of Prospero did not give a type II to type I transformation, but rather led to the loss of type II NBs via death or differentiation (Table 1), as previously reported for misexpression of Prospero in type I NBs [24]. We propose that these NBs are missing due to differentiation because we observe large cells with both nuclear Prospero and Dpn as well as large cells with just nuclear Prospero (Figure 3F-H), consistent with Prospero inducing downregulation of Dpn as the first step in differentiation. We conclude that misexpression of Prospero in type II NBs does not transform them to a type I NB identity.

### Prospero misexpression suppresses INP proliferation

**Figure 4:**
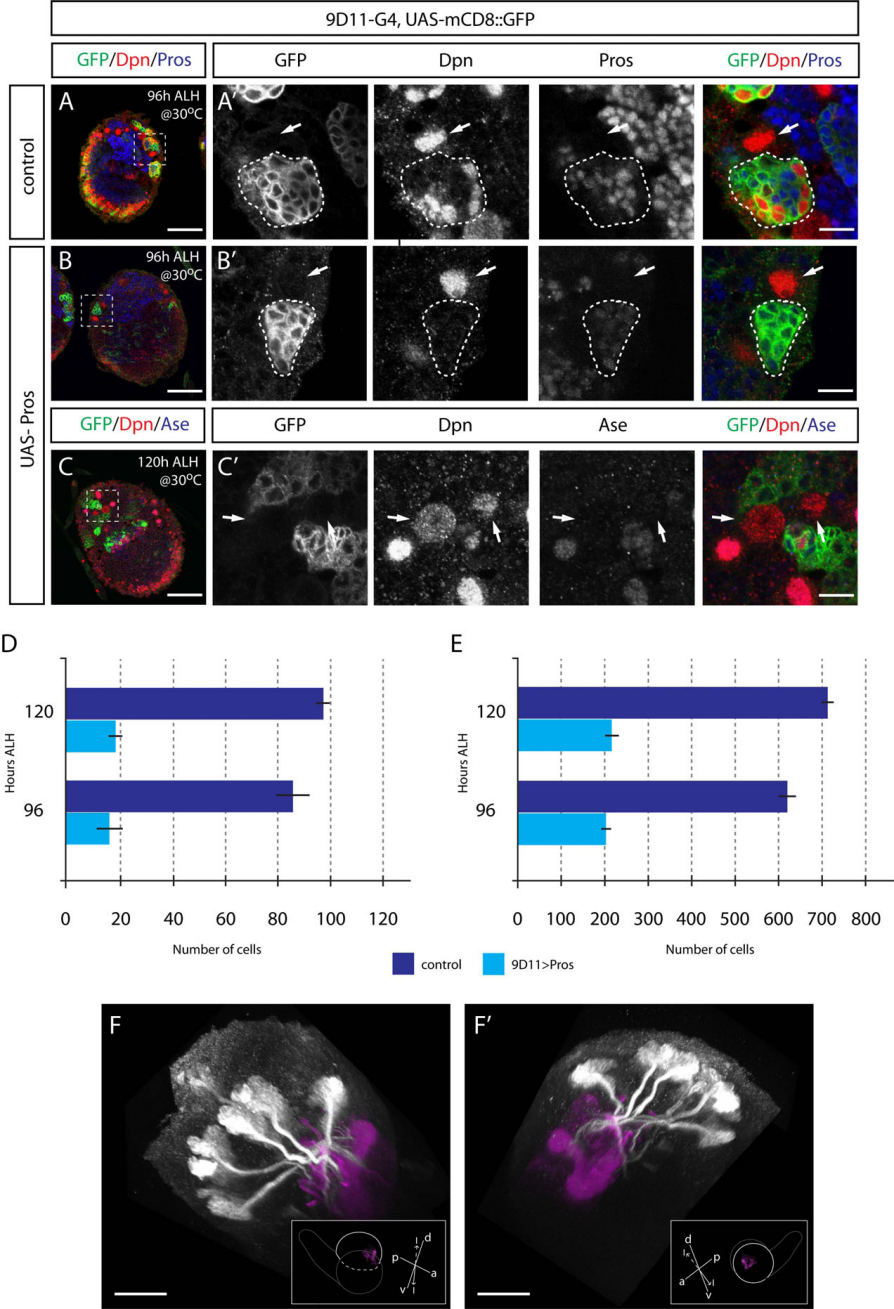
**Prospero misexpression suppresses INP proliferation**. **(A-C) **Confocal images of third instar larval brains expressing mCD8::GFP (A) or mCD8::GFP and Pros (B,C) under 9D11-Gal4 stained for indicated markers (white boxes). Low magnification images of single brain lobes are presented in (A-C), and higher magnification images of boxed areas are presented in (A'-C'), respectively. White outlines represent the GFP-labeled areas. Type II NB are indicated with white arrows; mature INPs are small Dpn+ cells in white outlined areas. **(D,E) **Histograms showing number of INPs (D) and total cells (E) labeled by mCD8::GFP driven by 9D11-gal4 in control and *prospero *overexpression brains at 96 and 120 h ALH. **(F,F') **Three-dimensional reconstruction of medial (F) and lateral (F') views of a 120 h ALH brain lobe expressing mCD8::GFP and UAS-Prospero under control of 9D11-Gal4. Type II lineages and their axonal projections are shown in white, and the mushroom body, visualized by FasII, is shown in magenta. The optic lobe is removed and the brain cropped for a clearer view. Brains are in the orientations shown in the insets, with imaged lobes indicated with a white dashed line and their mushroom bodies shown. Orientation: d, dorsal; v, ventral; p, posterior; l, lateral; m, medial. Scale bars: (A-C) 50 μM; (A'-C') 10 μM; (F,F') 40 μM.

Misexpression of Prospero in type II NBs and their progeny using the 19H09 driver resulted in many Prospero+ small progeny around the NB (Figure 3E), and a large reduction in the number of neurons generated by each type II NB (Figure 3B,C; Table 1). The reduction of clone size could be due to reduced proliferation of the parental NB or the INPs. To distinguish between an effect on the NB versus INPs, we used the INP-specific Gal4 line 9D11 to misexpress Prospero. We found no difference in NB numbers, but we observed a striking reduction in the number of INPs and total cells at both 96 h and 120 h ALH (Figure 4A-E; Table 2; 96 h ALH). We conclude that misexpression of Prospero does not affect type II lineage identity, but rather it suppresses INP proliferation, and that type II lineages are much larger than type I lineages, in part due to the absence of Prospero from the new-born INP progeny.

### 9D11 is expressed in a small subset of neurons in the adult brain that project to the fan-shaped body of the central complex

We and others have shown that although there are only 8 type II NBs among the approximately 100 central brain NBs, the type II NBs generate a disproportionately high percentage of the total neurons in the late larval brain [3-5]. We were curious to know if the shared developmental history of the type II neurons directs them to form a specific structure in the adult brain, or whether these neurons are dispersed throughout the adult brain. Recent work has shown that clones generated within type II NBs preferentially contribute to the central complex of the pupal brain [23], supporting a 'common function' model. The *Drosophila *central complex is a major neuropil in the adult brain that has been implicated in several behaviors, including locomotion, flight, and visual pattern memory [25-27], and consists of four interconnected substructures located on the midline of the protocerebrum: the protocerebral bridge (PB), the fan-shaped body (FB), the paired noduli (NO) and the ellipsoid body (EB). These neuropils are closely associated with the accessory areas, lateral accessory lobes (LAL; also known as ventral bodies) and bulbs (BUs; also known as lateral triangles) [28-30]. In addition, central complex neurons can be classified as either large-field or small-field. Large-field neurons link a single central complex substructure to regions outside the central complex; most project to one of the accessory areas. Small-field neurons are primarily intrinsic to the central complex, where they innervate a single substructure or link two to three substructures in a columnar fashion [29,30].

**Figure 5:**
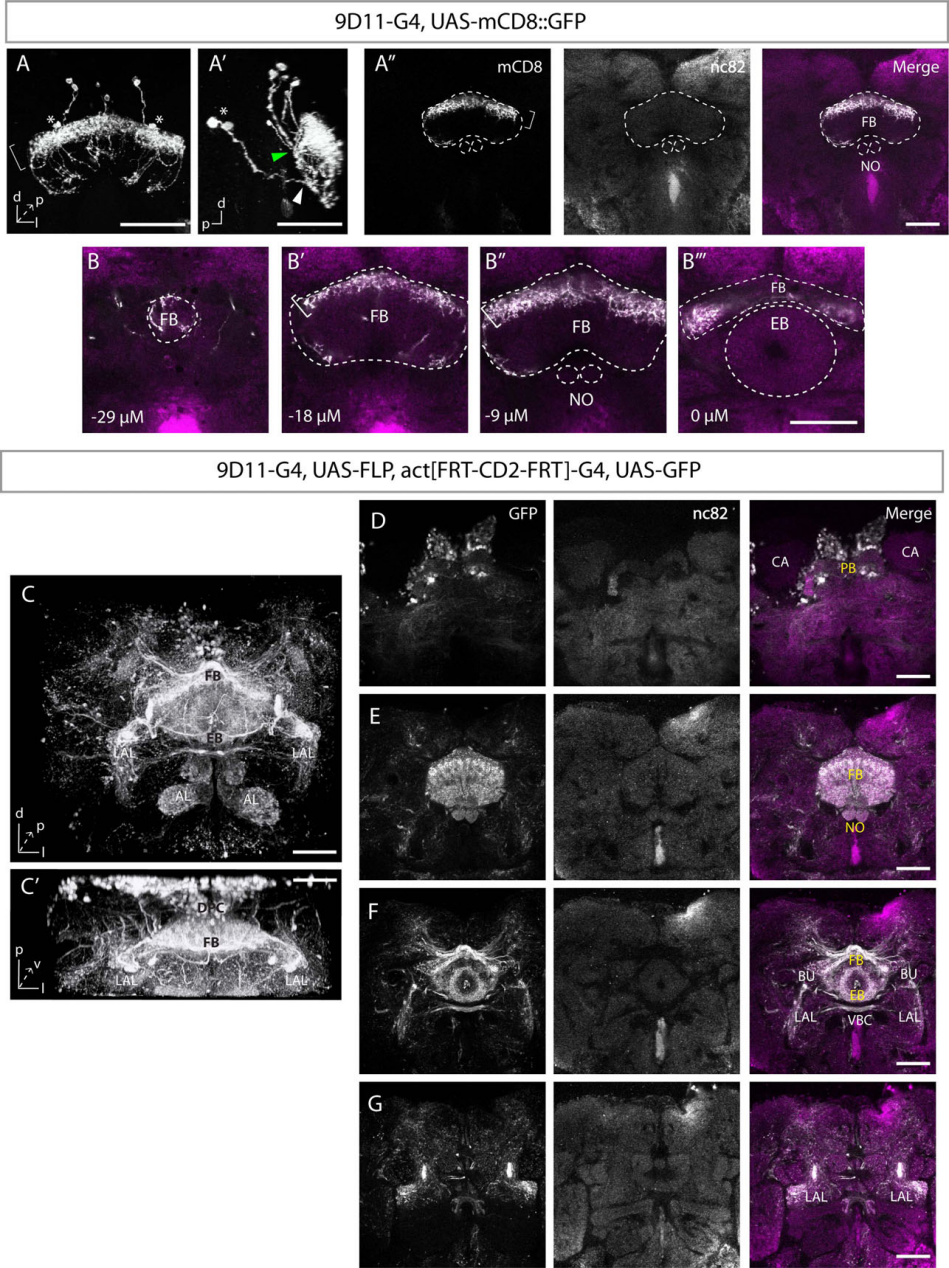
**Lineage tracing with 9D11 labels the adult central complex and associated regions**. **(A,B) **9D11 expression in the adult brain stained for mCD8 (white) and synaptic marker nc82 (magenta). (A,A') Frontal (A) and sagittal (A') views of the three-dimensional reconstruction of mCD8::GFP confocal z-stacks, close up on the FB. Three cell pairs are located dorsal to the FB while two cell pairs are more ventral at the level of the dorsal FB (asterisks). The projections from the dorsal cell pairs enter the dorsal FB at medial sites (green arrowhead) while the projections of ventral pairs enter the dorsal FB at more anterolateral sites (white arrowhead). See Additional file 5 for the three-dimensional reconstruction. (A'') Single frontal confocal section of the same brain at the level of the FB. (B-B''') Serial higher magnification frontal confocal sections of the central complex from posterior to anterior. Cell bodies are posterior to (B). White brackets indicate the dense dorsal layer of innervations at the FB. White outlines represent the neuropils visualized by nc82 staining (labeled). **(C-G) **Lineage tracing with 9D11-Gal4 in the adult brain stained for GFP (white) and nc82 (magenta). (C,C') Frontal (C) and dorsal (C') views of the three-dimensional reconstruction of GFP confocal z-stacks. (C) Most cell bodies in the posterior cortex were removed for easier viewing. (D-G) Serial frontal confocal sections of the same brain from posterior to anterior show labeling at the PB and cell bodies in the posterior brain (D), the FB and NO (E), the anterior FB, EB, LALs, and BUs (F), and the LALs and middle brain (G). CA, calx. VBC, ventral body commissure. See Additional file 6 for more representative stacks, Additional file 8 for high magnification images of the central complex, and Additional file 7 for the three-dimensional reconstruction. Orientation: d, dorsal; v, ventral; p, posterior; l, lateral. Scale bars: 40 μM.

To trace the projections of type II NB progeny, we used the INP-specific 9D11-Gal4 line to assay for adult brain expression directly, as well as to induce expression of a heritable genetic marker in INPs during larval stages and assay cell body position and axon projections in the adult brain. First, we observed that 9D11 was expressed in a small subset of neurons in the adult brain that projected to the FB region of the central complex (Figure 5A,B; Additional file 5). The cell bodies were located in the dorsal posterior complex medial to the mushroom body calyces and their projections entered the FB at different sites (Figure 5A,A') They formed a dense layer of arborizations at the top sections of the dorsal FB, and in a columnar fashion along the vertical staves throughout the rest of the FB (Figure 5A,B). The projections were confined to the FB and did not enter the NO or EB (Figure 5B-B'''). The position and projections of these neurons match the P3 or P4 small-field pontine neurons that are intrinsic to the FB [29,30]. Our observations are consistent with a previous study on the adult brain expression pattern of 9D11 [22]. We conclude that 9D11 is expressed in pontine neurons, small-field neurons of the adult FB [29,30]. These results are consistent with those showing type II lineages projecting to the central complex at pupal stages [23], but we can not definitely say that these neurons are derived from type II lineages solely based on adult 9D11 expression. Thus, we next turned to inducing permanent expression of GFP in the 9D11+ INP progenitors during larval stages, and assaying their position and projection in the adult brain.

### Lineage tracing of 9D11-expressing cells labels the central complex and associated regions in the adult brain

To identify the structures type II lineages contribute to adult brain, we crossed 9D11 to *UAS-FLP*, *actin[FRT-CD2-FRT]gal4*, *UAS-GFP *and induced FLP-out clones at larval stages to permanently express GFP in INPs and their neuronal progeny. We found that type II lineages primarily contribute to the central complex of the adult brain, as well as some optic lobe labeling due to 9D11 expression in this tissue.

A detailed analysis of the adult brain pattern revealed the majority of the labeled cell bodies in the dorsal posterior cortex (Figure 5; Additional file 6), similar to the few neurons that maintain 9D11 expression in the adult brain (previous section). Additional cell bodies were seen in the anterior cortex lateral to the anterior LAL and other areas (Additional file 6).

We next describe the adult brain axon projection patterns for the type II lineages, although the high density of labeling made it difficult to link axon projections to specific cell bodies. We observed labeling of all four central complex neuropils, the two central complex accessory areas and several other regions in the central brain (Figure 5C-G; Additional files 6 and 7).

#### Central complex: protocerebral bridge neuropil

The PB neuropil is the most posterior of the central complex and is divided into 16 segments [29]. The PB was diffusely labeled with its lateral edges showing slightly denser staining, and the segments were not distinguishable (Figure 5D; Additional file 8A; compare Additional file 6C to 6D for denser labeling of lateral PB). Several types of small-field neurons connect the PB to other central complex neuropils but we could not distinguish them by their dispersed projections in the PB. The projections we observed in other neuropils suggest that small-field types, such as ventral fiber system (VFS) and horizontal fiber system (HFS) neurons, which connect the PB to the FB, and pontine, pb-eb-no, and eb-pb-lal neurons are labeled (see sections below) [29].

#### Central complex: fan-shaped body neuropil

The FB is the largest structure in the central complex and is divided into several vertical staves and horizontal stratifications [29]. Small-field neurons, which typically have their cell bodies in the DPC, contribute largely to the vertical staves while large-field neurons, which are found in both the posterior and anterior cortex, form most of the horizontal strata [29]. The FB was heavily innervated throughout, revealing its vertical and horizontal layers (Figure 5E,F; Additional files 6D-H and 8B-D). A single horizontal layer in the dorsal FB was more heavily innervated than other sections (Figure 5E; indicated with yellow dashed lines in Additional file 8B-D). We also observed dense staining in tracts dorsal to the anterior FB that are connected to arborizations in the posterior superior medial protocerebrum (psmpr) and middle superior medial protocerebrum (msmpr) regions; these projections appear to connect to the LALs as well (Figure 5F; Additional file 6E-I). We propose that these tracts are part of the anterior commisure of the FB [29].

The cell bodies and the projection pattern of several small-field types match our observations in the FB and other central complex neuropils. These include VFS and HFS neurons that project along the vertical staves (Image 5 and 6 in [24]), pontine neurons that innervate all parts of the FB (Image 9 in [24]), fb-eb neurons that innervate two horizontal layers in the FB (Image 7 in [30]) and fb-no neurons that are restricted to few staves and horizontal layers (Image 11c,d in [24]). The cell bodies and the projection pattern of some large-field F neurons (fan-shaped neurons) also match our observations in the FB. The F*m*1 and F*m*3 subtypes (fan-shaped medial neurons) have cell bodies in the DPC, and F*l *subtypes (fan-shaped lateral neurons) are primarily in the anterior cortex ventrolateral to LALs. The F*m*1 and F*m*3 neurons project anterior to the FB then posterior through the EB canal to form arbors in the second ventral layer of FB, whereas F*l *neurons project to all layers of the FB [29]. Some F*l *neurons project through the anterior commisure and innervate the msmpr (Image 22 g in [24]). Another type of F*l *neuron, ExF*l*2 (an extrinsic fan-shaped neuron), has its cell body located in the DPC lateral to mushroom body calyces and forms arbors at psmpr before innervating a dorsal horizontal FB layer in a segmented fashion (Image 13 in [30]). These projections are remarkably similar to those made by type II-derived neurons, especially the tracts dorsal to anterior FB that are connected to arbors in the psmpr and msmpr (Additional file 6) and the dense segmented dorsal layer of innervations at the FB (Additional file 8, indicated with yellow dashed lines).

#### Central complex: ellipsoid body neuropil

The EB neuropil is anterior to the FB and can be divided into a posterior and anterior ring. The posterior EB is innervated by small-field neurons while large-field R neurons (ring neurons), which have cell bodies ventrolateral to the LALs in the anterior cortex, fill the anterior and the median parts of the EB in concentric rings [29,31]. However, certain R neurons are known to innervate only fragments of the EB, and ExR2, a rare extrinsic type of R neuron, is known to innervate the posterior EB only [29]. Parts of the EB were also innervated (Figure 5F; Additional file 6G,I). The posterior ring of EB was innervated in a ring-like fashion (dorsoposterior part in Additional file 8C and the middle ring in Additional file 8D); however, the more dorsoanterior parts were less innervated (Additional file 2E,F). The innervation of the anterior ring of the EB was weaker and found in a radial, evenly spaced fashion rather than a continuous ring (Additional file 8E,F). Projections through the EB canal were also observed (Additional file 8D-F, circle inside the anterior ring).

While the R neurons that project to fragments of EB could contribute to the staining of the posterior ring of EB, it is more likely generated by the small-field types such as fb-eb and pb-eb-no neurons or the rare ExR2 neuron [29], which mostly innervate the posterior ring of EB [29].

#### Central complex: noduli neuropil

The NO neuropil is ventral to the FB and is divided into three horizontal layers. Several small-field types innervate the NO [29,30]. The NO was also heavily innervated (Figure 5E; Additional file 6F,G). The three horizontal layers of the NO were revealed (Figure 2C) and the top layers were heavily innervated (Additional file 8C, arrows) [30]. This pattern matches the projections of fb-no and pb-eb-no small-field neurons, which innervate only the dorsal segments of the NO [29].

#### Central complex: accessory areas

Few small-field neurons project to small regions of LALs, while many large-field neurons innervate the whole LAL neuropil [29]. BUs are also innervated by both small-field and large-field neurons and they are connected to the contralateral LALs [29]. In addition to the four central complex neuropils, the LAL and BU accessory areas were also labeled (Figure 5F,G; Additional file 6G-K). There were widespread arborizations in the LALs, including the ventral body commissure that connects LALs across the midline (Figure 5F). Small regions in the lateral sides of the dorsoanterior LALs, bound dorsally by the mushroom body medial lobes and ventrally by the antennal lobes (ALs), were innervated heavily (Figure 5G; Additional file 8J,K). We also observed labeling of BUs and connections between BUs and ipsilateral LALs (Figure 3F). The extensive labeling of LALs accompanied with dense staining of small regions and the labeling in BUs is consistent with the notion that large-field types, like F*l *neurons, and small-field types, such as eb-pb-lal, HFS, and pb-eb-bu neurons, are derived from type II lineages [29]. We conclude that type II lineages contribute to all central complex neuropils and accessory areas in the adult brain.

Outside the central complex, we observed dense innervation in a region that lies dorsal to the LALs, posterior to the mushroom body medial lobes, and lateral to the anterior EB (Figure 5G; Additional file 6). The central and anterior parts of medial protocerebrum were also labeled (Additional file 6F-M). Interestingly, projections were observed in the mushroom body vertical and medial lobes (Additional file 6L,M) as well as specific glomeruli in the AL (Additional file 6J-M). The labeling we observe outside the central complex could be connections between the central complex and other brain regions or non-central complex neurons made in type II lineages.

## Discussion

The recent identification of the type II lineages containing transit amplifying intermediate progenitors provides an important new model for investigating progenitor self-renewal and differentiation [3-5,14]. However, we know little about their development, cell biology, gene expression, and functional importance in the *Drosophila *central nervous system. This is primarily due to a lack of genetic tools and markers that are specifically expressed in type II NBs and/or INPs. Here we characterize the 19H09-Gal4 line expressed in type II NBs, and the 9D11-Gal4 line expressed in INPs but not their parental type II NBs. Using 19H09 we show that Ase is upregulated before Dpn during INP maturation. Using both lines, we show that Prospero misexpression regulates proliferation but not identity within type II lineages. And using 9D11 we permanently label the majority of type II-derived neurons to show they are major contributors to the adult central complex brain region.

### 19H09 and 9D11 as tools to understand brain development and function

The 19H09-Gal4 and 9D11-Gal4 lines can also be used to monitor the development of type II NBs and INPs in different mutant backgrounds to help clarify the origin of a mutant phenotype. For example, early studies on tumor suppressor genes showed increases in global brain NB numbers; for some of these mutants (for example, *brat*, *numb*) we know now that the phenotype arises specifically within the type II lineages [5]. The 19H09-Gal4 and 9D11-Gal4 lines can also be used to drive UAS-RNAi, UAS-GFP constructs to test the role of any gene within these lineages. In addition, because these lines are made from defined enhancer fragments driving Gal4 placed into a specific attP site in the genome, it is easy to generate different transgenes with precisely the same expression pattern. Some future uses would be: using 19H09-FLPase to generate mutant clones or MARCM genetic screens in type II lineages; using 9D11 to drive expression of uracil phosphoribosyltranferase [32] to isolate RNA from INP sublineages; or using 9D11-grim to ablate specifically type II neurons to determine their role in larval or adult behavior.

### The role of Prospero in type I and type II NB lineages

We have used the 19H09 and 9D11 lines to show that misexpression of Prospero can suppress proliferation within type II NBs and INPs without altering NB identity. As 19H09 is expressed only during the late larval stages, Prospero misexpression with 19H09 clearly distinguishes the effects of Prospero on NB proliferation from its effects on NB fate specification, which occurs in the embryonic stages. Misexpression with both 19H09 and 9D11 lead to a reduction in the number of INPs and neurons made by each type II NB. This reduction is unlikely to be due to an effect on the parental type II NBs, such as slowed down cell cycle or compromised NB survival, for the following reasons: first, low levels of ectopic Prospero are cytoplasmic in type II NBs, where Prospero has no known function; second, ectopic Prospero does not transform type II lineages to a type I identity based on the failure to upregulate *ase *expression; and third, misexpression of Prospero with both 9D11 and 19H09 give similar phenotypes, yet 9D11 is not expressed in type II NBs. We suggest that the reduction of clone size is due to an effect in the INP cell type. Possible mechanisms include INP apoptosis, INP cell cycle lengthening, premature cell cycle exit, or transforming INPs into central brain type II GMCs, which generate lineages with bifurcated axon fascicles. While we could not distinguish between these possibilities, we can tentatively exclude the mechanism of a transformation of INP to central brain type I GMC identity because the neurons still retained their ability to form bifurcated axon fascicles (Figure 4F; Additional file 4), which are not a feature of central brain type I GMCs.

Type II NBs lack both Ase and Prospero, whereas type I NBs contain both proteins. Yet only misexpression of Ase can transform type II into type I NBs ([4] and this work), suggesting that Ase is sufficient to upregulate *prospero *expression in NBs. However, loss of Ase does not transform type I NBs into type II NBs [5], so there must be additional factors promoting the expression of Prospero in type I NBs. The analysis of gene expression differences between type I and II NBs would be one way of uncovering genes that control the difference between them.

### The contribution of type II lineages to the adult brain

Lineage-tracing of INP-derived neurons shows that type II lineages make major contributions to all aspects of the central complex of the adult brain, as well as the BU and LAL accessory structures, including both small-field and large-field neurons [29]. Central complex neurons derived from type II lineages likely include several small-field types, such as VFS, pontine, fb-eb, fb-no, and pb-eb-no neurons, and, to a lesser extent, large-field types, such as F neurons, including F*m*, F*l *and ExF*l *subtypes and some extrinsic R neurons. A recent study found that type II NB clones in the pupal brain projected to the PB, FB and NO regions, with some projections forming restricted arbors at the PB and innervating domains of the FB and NO, while others made widespread arborizations outside the central complex [23]. Our data showing labeling of the majority of type II neuronal progeny are consistent with those of [23], and complementary to these data: while we do not have the resolution to link cell bodies with axon projections, we are able to provide a more comprehensive view showing that type II lineages contribute to all central complex neuropils and accessory areas in the adult brain. Future studies that selectively ablate different spatial or temporal cohorts of type II neurons will be necessary to determine if all type II-derived neurons share a common function.

Although a large subset of central complex neurons derive from type II lineages, there are clearly some central complex neurons that originate from type I NBs or embryonic type II lineages. For example, we do not see projections that match those of the well-characterized large-field R neurons (R1 to R4) [29,31]. It is not clear which small-field types are not derived from type II lineages as they are difficult to distinguish. However, it is clear that the type II lineages do not make up the entire central complex so there must be contributions from type I lineages as well.

Outside the central complex, we observed labeling of the region-specific staining of both the mushroom body and ALs; staining in the ALs was restricted to a subset of glomeruli. These could be novel connections from the central complex to the mushroom body and ALs formed by large-field or poorly understood extrinsic small-field neurons [29], or the projections of non-central complex neurons labeled by 9D11. Previous studies have revealed no direct connection between central complex and mushroom bodies or between LALs and ALs, and very few connections from LALs to mushroom bodies [29,33]. The type II projection patterns from larval and pupal brains suggest that the lineages are not dedicated to a single neuropile center, which is consistent with type II lineages giving rise to non-central complex neurons as well. We also observed labeling of large regions in the protocerebrum outside the central complex. However, it was not possible to distinguish whether they were connected to the central complex or its accessory areas. Another caveat to our analysis is that 9D11 is also expressed in the larval optic lobes, and indeed we observed labeling in the adult optic lobes (Additional file 6, R-R'''). We could not distinguish the projections from these cells from those of the central brain cell bodies due to dense staining. Analysis of 1,200 Golgi-impregnated brains revealed direct connections between optic lobes and the BU neuropil, but not to the other central complex neuropils that we find labeled [29]. This suggests that most if not all central complex labeling is due to type II-derived neurons.

In addition to using 9D11 to lineage trace the contribution of larval-derived type II neurons to the adult brain, we also detected maintained expression of 9D11 in a small subset of adult neurons, which are likely to be P3 or P4 small-field pontine neurons, which are also detected by the Gal4 line NP2320 [30]. Thus, the 9D11 line, and others with similarly specific adult expression patterns, should be useful for future studies using TU-tagging to transcriptionally profile neuronal subsets [32], GRASP to identify pre/post-synaptic partners [34], or for expression of optogenetic modulators of neuronal activity to determine the role of specific neurons in behavior [35].

Our characterization of type II lineages suggests that as a group the type II NBs produce a wide variety of neuronal subtypes. This neural diversity can be achieved spatially if each type II NB generates just one or two types of neurons; this model is supported by clonal data showing that each type II NB produces neurons with distinct axon projection patterns [23]. In addition, temporal identity could generate further neuronal diversity as seen in type I NB lineages [36]. This model is supported by clonal analysis of a small central complex sublineage in the adult brain, which has revealed temporally distinct neuronal fates [37]. Finally, hemilineages could provide a final doubling of neuronal diversity, in which each sibling neuron derived from a single GMC takes either an 'A' or a 'B' cell fate [13]. The fact that bifurcating axon projections are seen even in the highly sparse type II lineages following Prospero overexpression is consistent with GMCs producing A/B neurons that have different fasciculation patterns. In the future, it will be important to determine the birth-order and identities of neurons in each type II lineage and the mechanisms that regulate spatial and temporal neural fate specification in these lineages.

## Materials and methods

### Fly stocks

Fly stocks were: *FRTG13*, *UAS-mcd8::GFP *(Bloomington Stock Center); *UAS-nls::GFP *(Bloomington Stock Center); *worniu-Gal4 *[38]; *9D11-Gal4 *[22]; *19H09-Gal4 *(G Rubin, unpublished); *UAS-prosL *[39] (F Matsuzaki, unpublished); *Act[FRT-CD2-FRT]-Gal4*, *UAS-GFP *(gift from Bruce Edgar) crossed to *UAS-FLP/CyO *(Bloomington Stock Center).

### Tissue preparation and immunohistochemistry

Larval brains were dissected in Schneider's medium (Sigma, St Louis, MO, USA); fixed in 100 mM Pipes (pH 6.9), 1 mM EGTA, 0.3% Triton X-100, and 1 mM MgSO_4 _containing 4% formaldehyde for 25 minutes; washed 30 minutes in phosphate-buffered saline (PBS) containing 0.3% Triton X-100 (PBS-T); washed 30 minutes in PBS-T with 1% bovine serum albumin (PBS-BT); and incubated with primary antibodies in PBS-BT overnight at 4°C. Afterwards, brains were washed 1 h in PBS-BT, incubated with secondary antibodies for 2 h and washed 1 h in PBS-T.

Adult females 3 to 10 days old were anesthetized on ice and dissected immediately in ice-cold PBS (dissection time per brain approximately 4 minutes). Brains were fixed in PBS with 4% formaldehyde for 25 minutes; washed 10 minutes in PBS containing 1% Triton X-100 (PBT) three times and blocked with PBT containing 5% normal-goat serum (Vector Laboratories, Burlingame, CA, USA) prior to incubation with primary antibodies in PBT overnight at 4°C. Afterwards, brains were washed 10 minutes in PBT three times, incubated with secondary antibodies for 2 h and washed 10 minutes in PBT three times.

Primary antibodies were rat Dpn monoclonal (1:1), rabbit Ase (1:2,000), mouse Prospero monoclonal (purified MR1A, 1:1,000), rabbit GFP (1:500; Molecular Probes, Eugene, OR, USA), mouse GFP (1:500; Molecular Probes), chicken GFP (1:500; Aves Laboratories, Tigard, OR, USA), rat mCD8 (1:150; Invitrogen, Eugene, OR, USA), mouse Fasciclin II (1:100; Developmental Studies Hybridoma Bank), mouse nc82 (1:10; Developmental Studies Hybridoma Bank), and mouse Dlg (1:100). Secondary antibodies were from Molecular Probes (Eugene, OR, USA) and diluted at 1:500 in PBS-BT or PBT for larval and adult brains respectively.

### Histology and imaging

Brains were mounted in Vectashield mounting medium (Vector Laboratories). Images were captured with a Biorad Radiance or Zeiss700 confocal microscope with a z-resolution of 1.0 (for three-dimensional reconstructions) or 1.5 microns and processed in ImageJ (NIH, Bethesda, MD, SUA) and Photoshop CS3 (Adobe, San Jose, CA, USA). Figures were made in Illustrator CS3 (Adobe). Three-dimensional brain reconstructions and movies were generated using Imaris software (Bitplane, Zurich, Switzerland).

## Abbreviations

aEB: anterior ring of EB; Aimpr: anterior inferior medial protocerebrum; AL: antennal lobe; ALH: after larval hatching; Ase: Asense; Asmpr: anterior superior medial protocerebrum; Brat: Brain tumor; BU: bulb; CA: calx; CX: central complex; DM: dorsomedial type II lineage; DPC: dorsoposterior complex; Dpn: Deadpan; EB: ellipsoid body; eb-pb-lal: neuron connecting EB to PB to LAL; ExF: extrinsic fan-shaped neuron; ExR: extrinsic ring neuron; F: fan-shaped neuron; FB: fan-shaped body; fb-eb: neuron connecting FB to EB; fb-no: neuron connecting FB to NO; F*l*: fan-shaped lateral neuron; F*m*: fan-shaped medial neuron; GC: great commisure; GFP: green fluorescent protein; GMC: ganglion mother cell; HFS: horizontal fiber system; INP: intermediate neural progenitor; LAL: lateral accessory lobe; Milpr: middle inferior lateral protocerebrum; Mimpr: middle inferior medial protocerebrum; msmpr: middle superior medial protocerebrum; NB: neuroblast; NO: noduli; P: pontine; PB: protocerebral bridge; pb-eb-bu: neuron connecting PB to EB to BU; pb-eb-no: neuron connecting PB to EB to NO; PBS: phosphate-buffered saline; pEB: posterior ring of EB; psmpr: posterior superior medial protocerebrum; R: ring neuron; VBC: ventral body commisure; VFS: ventral fiber system; Vlpr: ventrolateral protocerebrum.

## Competing interests

The authors declare that they have no competing interests.

## Authors' contributions

OAB carried out all the experiments and participated in the design of the study. JQB participated in the characterization of Gal4 lines and the design of the study. MD participated in Prospero misexpression experiments. CQD conceived of the study. OAB and CQD wrote the manuscript. All authors read and approved the final manuscript.

## Supplementary Material

Additional file 1 **19H09-Gal4 labels a subset of type II neuroblasts and their progeny**. Additional files 1 to 4 are three-dimensional reconstruction movies of confocal stacks of late larval (120 h ALH) brain lobes stained for GFP (white) visualizing axon projections and FasII (magenta) visualizing the mushroom body. Optic lobes have been removed and brains cropped for easier viewing. At the beginning of the movies, the brains are aligned on the neuraxis and shown in medial view: dorsal is up, ventral is down, posterior is left (mushroom body calyx), and anterior is right (mushroom body dorsal lobe). 19H09 expressing mCD8::GFP, type II lineages and their projections are shown in white, type I lineages and their projections in red. Additionally, a subset of neurons that project to the mushroom body are visualized by 19H09. Type II lineages make bifurcating projections towards different parts of the brain, including the interhemispheric commisure. Click here for file

Additional file 2 **9D11 specifically labels INPs and their progeny within the central brain**. 9D11 expressing mCD8::GFP, INPs and their progeny are shown in white. 9D11 is strongly expressed in six medial type II lineages and weakly in two lateral type II lineages beginning at 96 h ALH. Again, bifurcating projections towards different parts of the brain are observed from type II lineages. All medial type II lineages project a subset of their axons to the interhemispheric commisure, especially towards the DPC. Descending ipsilateral projections are also observed. Click here for file

Additional file 3 **Prospero misexpression in type II NBs reduces lineage size but does not induce type I NB identity**. 19H09 expressing mCD8::GFP and Prospero, type II lineages and their projections are shown in white. While Prospero misexpression reduces the lineage size (compared to Additional file 1), it does not induce type I NB identity as type II lineages make bifurcating projections towards different parts of the brain Click here for file

Additional file 4 **Prospero misexpression suppresses INP proliferation**. 9D11 expressing mCD8::GFP and Prospero, INPs and their progeny are shown in white. While Prospero misexpression reduces the number of INPs and neurons, it does not induce a type I GMC identity in INPs as bifurcating projections towards different parts of the brain are observed from type II lineages. Click here for file

Additional file 5 **9D11 is expressed in a small subset of pontine neurons that project to the FB in the adult brain**. Three-dimensional reconstruction movie of confocal stacks of an adult brain expressing mCD8::GFP under control of 9D11, stained for mCD8 (white), close-up on the FB. At the beginning of the movie, the brain is in the frontal view: anterior is towards the viewer, posterior is towards the screen, dorsal is up, ventral is down, and lateral is towards left and right. Approximately a dozen cell bodies are visualized by 9D11 and their projections enter the FB at different sites to the FB: the ventral cell pairs enter the FB at its lateral edges, which are also more anterior to the entry sites of dorsal cell pairs. Click here for file

Additional file 6 **Lineage tracing with 9D11 labels the adult central complex and associated regions**. **(A-M) **Three-dimensional reconstruction of the brain presented in Figure 5C-G is shown in sagittal view (A) and serial frontal confocal sections through the same brain are shown from posterior to anterior (B-M). The positions of several sections are indicated in (A), the z-position of each confocal section relative to M are also shown in the right panels. (B) The majority of cell bodies can be seen in the DPC medial to mushroom body calyces. (K) Connections between the ALs are labeled. (L,M) Specific glomeruli of the ALs are labeled. White and yellow brackets indicate labeling at the mushroom body medial and vertical lobes, respectively. The latter was innervated more heavily. The dorsal parts of mushroom body vertical lobes, which were innervated sparsely, are indicated with asterisks. **(N-P) **Serial low magnification frontal confocal sections through another brain of the same genotype are shown with their relative z-positions to (P) showing the locations of labeled cell bodies. Cell bodies were found in the posterior cortex (N), including the DPC and areas ventral and ventrolateral to mushroom body calyces, middle inferior lateral protocerebrum (milpr) and ventrolateral protocerebrum (vlpr) regions (O,P), the latter lateral to anterior LAL, regions next to the mushroom body vertical lobes (P), and around the optical tubercule (not shown). **(R) **Z-projection image of serial low magnification frontal confocal sections through the anterior brain of the same genotype showing labeling in optic lobes. **(R'-R''') **Single confocal sections with their relative z-positions to (R'). Abbreviations are listed in the Abbreviations section. Scale bars: 40 μM. Click here for file

Additional file 7 **Lineage tracing of 9D11-expressing cells labels the central complex and associated regions in the adult brain**. Three-dimensional reconstruction movie of confocal stacks of an adult brain expressing GFP in INP progeny, stained for GFP (white).The central brain is shown. At the beginning of the movie, the brain is in the frontal view: anterior is towards the viewer, posterior is towards the screen, dorsal is up, ventral is down, and lateral is towards left and right. The majority of cell bodies are in the posterior cortex. All central complex neuropils, except the PB, which is occluded by the cell bodies, and the accessory area LALs are distinguishable in the movie. The anterior commisure of the FB and other parts of the brain, including large areas in the medial protocerebrum and specific glomeruli in the ALs, are innervated as well. Click here for file

Additional file 8 **High magnification images of the labeling at the central complex**. **(A-F) **Serial high magnification frontal confocal sections of the central complex of the adult brain presented in Figure 3C-G and Additional file 6 from posterior to anterior. The z-position of each confocal section relative to (F) are also indicated. White outlines represent neuropils visualized by nc82 staining. Yellow dashed lines indicate the dense layer of innervations at the dorsal FB. See text for details. Abbreviations are listed in the Abbreviations section. Scale bars: 20 μM. Click here for file
