# Supplementary figures and images for: Drosophila type II neuroblast lineages keep Prospero levels low to generate large clones that contribute to the adult brain central complex
Source: Neural Dev. 2010 Oct 1;5:26. doi: 10.1186/1749-8104-5-26 (PMC2958855; doi:10.1186/1749-8104-5-26)

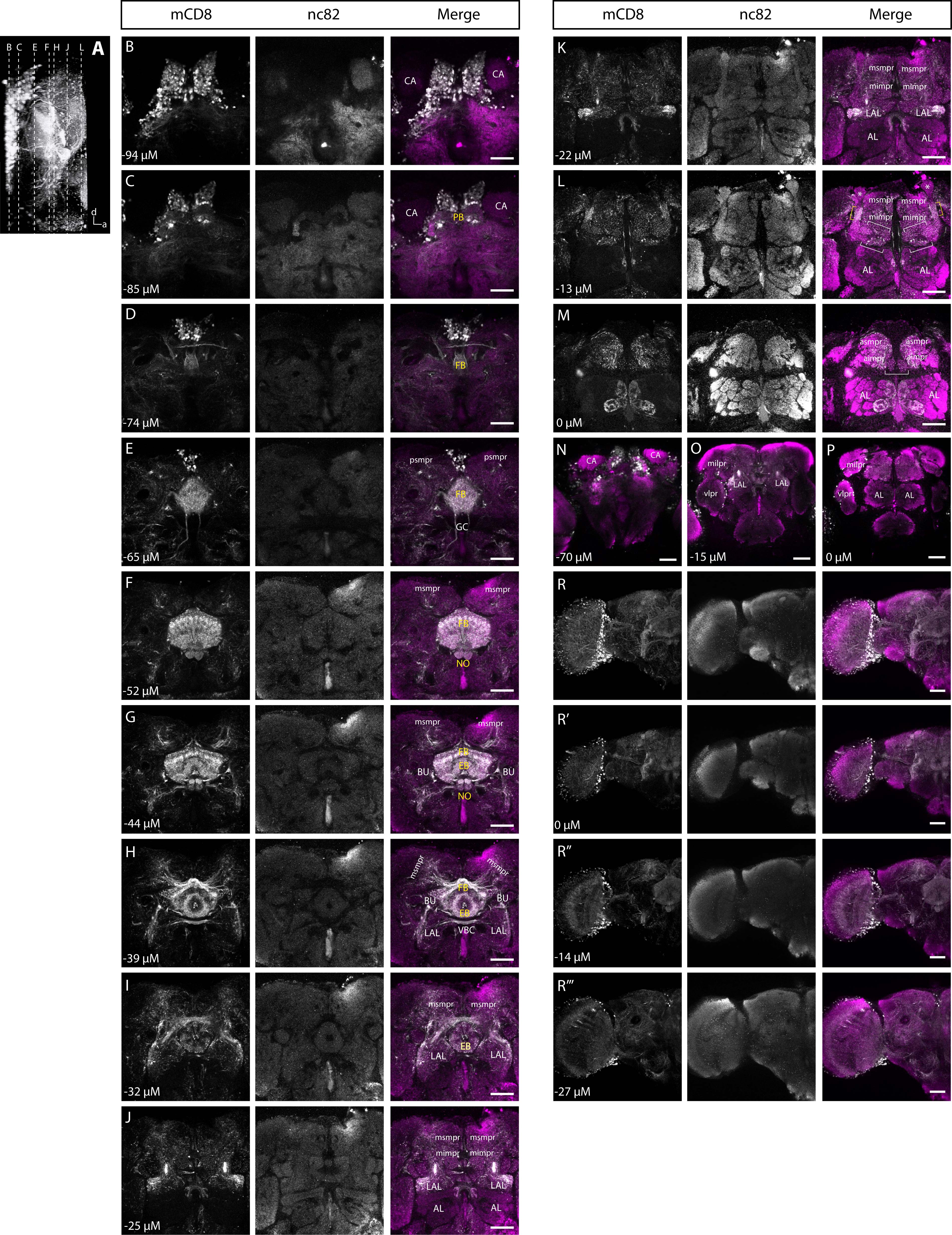

Supplement: Additional file 6 — Lineage tracing with 9D11 labels the adult central complex and associated regions. (A-M) Three-dimensional reconstruction of the brain presented in Figure 5C-G is shown in sagittal view (A) and serial frontal confocal sections through the same brain are shown from posterior to anterior (B-M). The positions of several sections are indicated in (A), the z-position of each confocal section relative to M are also shown in the right panels. (B) The majority of cell bodies can be seen in the DPC medial to mushroom body calyces. (K) Connections between the ALs are labeled. (L,M) Specific glomeruli of the ALs are labeled. White and yellow brackets indicate labeling at the mushroom body medial and vertical lobes, respectively. The latter was innervated more heavily. The dorsal parts of mushroom body vertical lobes, which were innervated sparsely, are indicated with asterisks. (N-P) Serial low magnification frontal confocal sections through another brain of the same genotype are shown with their relative z-positions to (P) showing the locations of labeled cell bodies. Cell bodies were found in the posterior cortex (N), including the DPC and areas ventral and ventrolateral to mushroom body calyces, middle inferior lateral protocerebrum (milpr) and ventrolateral protocerebrum (vlpr) regions (O,P), the latter lateral to anterior LAL, regions next to the mushroom body vertical lobes (P), and around the optical tubercule (not shown). (R) Z-projection image of serial low magnification frontal confocal sections through the anterior brain of the same genotype showing labeling in optic lobes. (R'-R''') Single confocal sections with their relative z-positions to (R'). Abbreviations are listed in the Abbreviations section. Scale bars: 40 μM. [file 1749-8104-5-26-S6.JPEG]

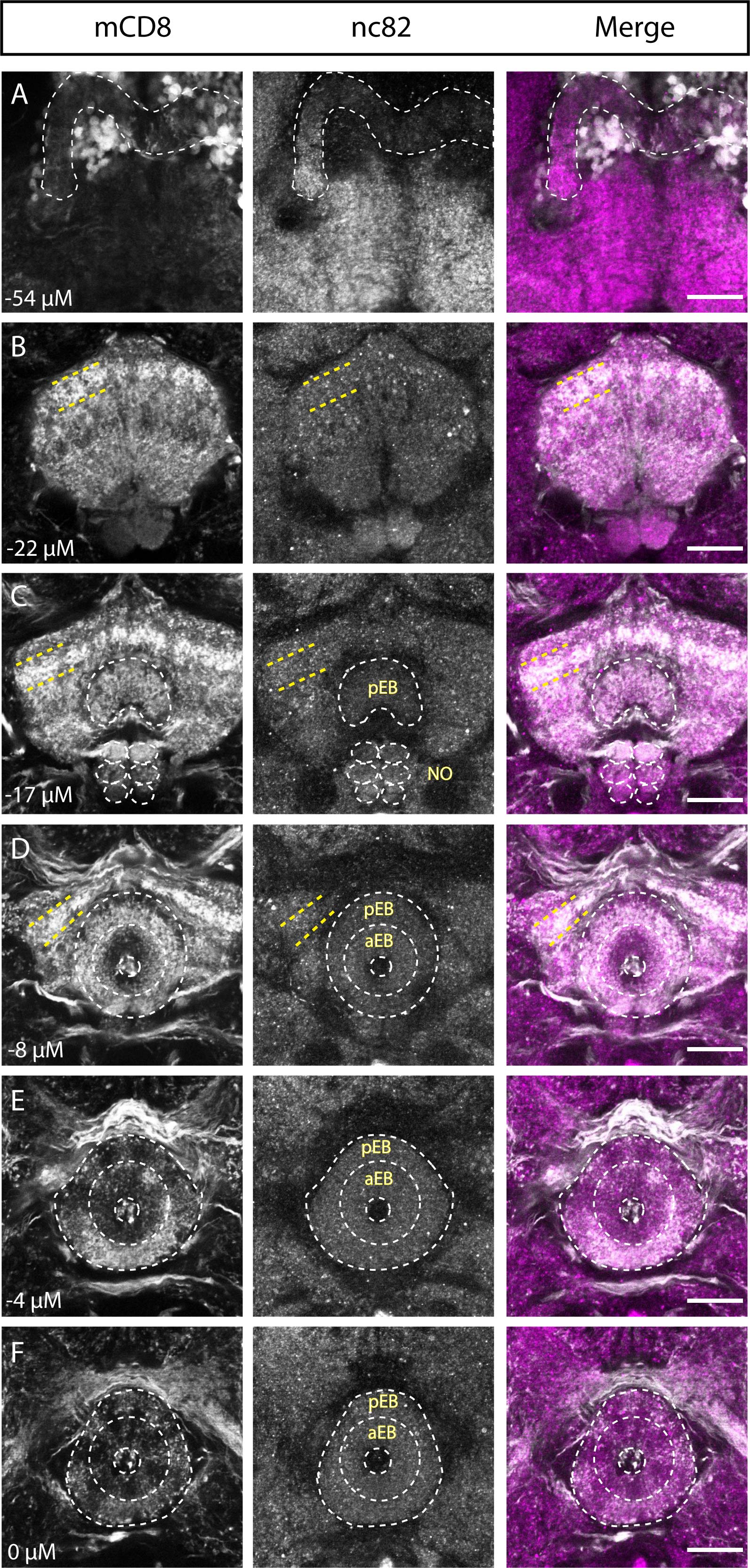

Supplement: Additional file 8 — High magnification images of the labeling at the central complex. (A-F) Serial high magnification frontal confocal sections of the central complex of the adult brain presented in Figure 3C-G and Additional file 6 from posterior to anterior. The z-position of each confocal section relative to (F) are also indicated. White outlines represent neuropils visualized by nc82 staining. Yellow dashed lines indicate the dense layer of innervations at the dorsal FB. See text for details. Abbreviations are listed in the Abbreviations section. Scale bars: 20 μM. [file 1749-8104-5-26-S8.JPEG]
